# The Chemokine CCL3 Regulates Myeloid Differentiation and Hematopoietic Stem Cell Numbers

**DOI:** 10.1038/s41598-018-32978-y

**Published:** 2018-10-02

**Authors:** Rhonda J. Staversky, Daniel K. Byun, Mary A. Georger, Brandon J. Zaffuto, Alexandra Goodman, Michael W. Becker, Laura M. Calvi, Benjamin J. Frisch

**Affiliations:** 1 Department of Medicine Hematology/Oncology Division University of Rochester School of Medicine and Dentistry, Rochester, NY USA; 2 Department of Medicine Endocrine Division University of Rochester School of Medicine and Dentistry, Rochester, NY USA; 3 Wilmot Cancer Institute, University of Rochester School of Medicine and Dentistry, Rochester, NY USA; 4 Center for musculoskeletal research, University of Rochester School of Medicine and Dentistry, Rochester, NY USA

## Abstract

The chemokine CCL3 is frequently overexpressed in malignancies and overexpression leads to microenvironmental dysfunction. In murine models of chronic myelogenous leukemia (CML), CCL3 is critical for the maintenance of a leukemia stem cell population, and leukemia progression. With CCL3 implicated as a potentially viable therapeutic target, it is important to carefully characterize its role in normal hematopoietic homeostasis. CCL3^−/−^ mice were used to evaluate the role of CCL3 in regulating hematopoietic stem and progenitor cell (HSPC) populations. CCL3^−/−^ mice had loss of mature myeloid populations, while myeloid progenitors and HSPCs were increased, and microenvironmental populations were unchanged. These data show that CCL3 promotes myeloid lineage differentiation and the size of the HSPC pool independent of the supportive bone marrow microenvironment. Our results demonstrate a previously unrecognized role of CCL3 in the maintenance of homeostatic hematopoiesis that should be evaluated when targeting CCL3 signaling for the treatment of hematologic malignancy.

## Introduction

The bone marrow microenvironment (BMME) is comprised of a complex network of cellular and molecular components that regulate hematopoiesis and hematopoietic stem cells (HSCs). The BMME contributes to the initiation and progression of hematologic malignancies such as chronic myelogenous leukemia (CML), acute myelogenous leukemia (AML), and myeloproliferative neoplasms (MPN)^1–3^. One mechanism that has been identified as a driver of BMME dysfunction is the inflammatory chemokine CCL3.

CCL3 is a chemotactic factor, signaling through the chemokine receptors CCR1 and CCR5 to recruit lymphocytes to sites of infection, and CD8+ T-cells to active lymph nodes^4,5^. In hematologic malignancies CCL3 is a well-known mechanism of BMME disruption in multiple myeloma, both activating osteoclastic bone resorption, and inhibiting bone forming osteoblastic cells^6^. We have previously identified CCL3 as an abundant leukemia-derived factor in a murine model of blast crisis chronic myelogenous leukemia (bcCML) that also displayed a profound loss of mature osteoblastic cells in the BMME^7^. CCL3 has also been implicated as a factor leading to microenvironmental dysfunction in a variety of malignancies including myelodysplastic syndrome (MDS), metastatic disease of renal cell carcinoma, and B-Cell lymphomas^8–11^.

In a murine model of CML, malignant cells that do not express CCL3 cannot maintain a leukemia stem cell (LSC) population and consequently do not give rise to leukemic disease^12^. In addition, when a mutant allele of the gene *Ptpn11* that is associated with juvenile myelomonocytic leukemia (JMML) is homozygously expressed in murine osteoprogenitors mice develop a myeloproliferative neoplasm reminiscent of JMML. The murine disease correlates with high levels of CCL3 expression and is reversed with the administration of CCL3 receptor antagonists^13^. In human AML approximately 75% of patients exhibit elevated expression of CCL3^7^, and overexpression of CCL3 in Nup98/HoxD13 expressing pre-leukemic cells leads to transformation to AML^14^. These studies confirm an essential role for CCL3 in the emergence and maintenance of LSCs in these murine models of myelogenous leukemias. Therefore, CCL3 is an appealing therapeutic target in hematologic malignancies.

The potential for translation of CCL3 targeting is further supported by the prior development of small molecule inhibitors of the CCL3 receptors CCR1 and CCR5. Multiple CCR1 inhibitors have been developed for the treatment of a variety of diseases such as rheumatoid arthritis and multiple sclerosis, and, having proceeded through phase 1 and 2 clinical trials, have been found safe for use in humans^15^. Further, the CCL3 receptor CCR5 is a coreceptor for the cellular entry of human immunodeficiency virus (HIV)^16^, and the FDA has approved the drug maraviroc, a small molecule CCR5 antagonist, for the treatment of HIV infection^15^. Since leukemia initiating cells share a number of characteristics with normal stem cells, such as increased self-renewal and a more quiescent state, targeting of LIC may also impact HSCs. In fact, the role of CCL3 in support of hematopoiesis is controversial. CCL3 was previously demonstrated to inhibit hematopoietic progenitor proliferation in colony forming assays^17,18^, to enhance myelopoiesis *in vitro*^19^, and to be dispensable for HSC maintenance *in vivo*^20^. To determine the necessity of CCL3 for maintenance of normal hematopoiesis we performed a comprehensive *in vivo* analysis of homeostatic hematopoiesis and the BMME in CCL3 knockout (CCL3^−/−^) mice^21^.

## Results

### Absence of CCL3 signaling leads to altered myeloid differentiation

**Figure 1:**
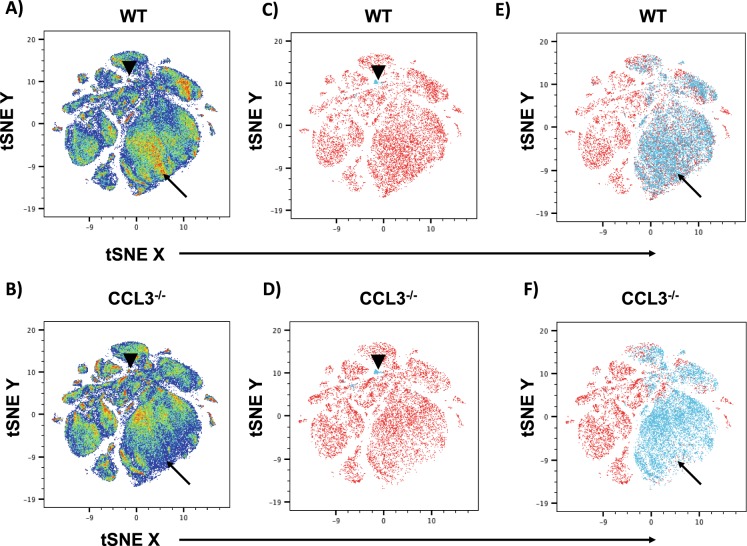
CCL3 regulates HSPCs and myeloid cells in the bone marrow. CyTOF data was analyzed using the tSNE algorithm. CyTOF data was collected separately from the marrow of 3 separate WT (**A**) and CCL3^−/−^ (**B**) mice and concatenated. The LSK cell population, denoted by arrow heads, is superimposed onto WT (**C**) and CCL3^−/−^ (**D**) tSNE plots. The CD11b + B220-CD2-Ter119- myeloid cell population, denoted by arrows, is superimposed onto WT (**E**) and CCL3^−/−^ (**F**) tSNE plots.

**Figure 2:**
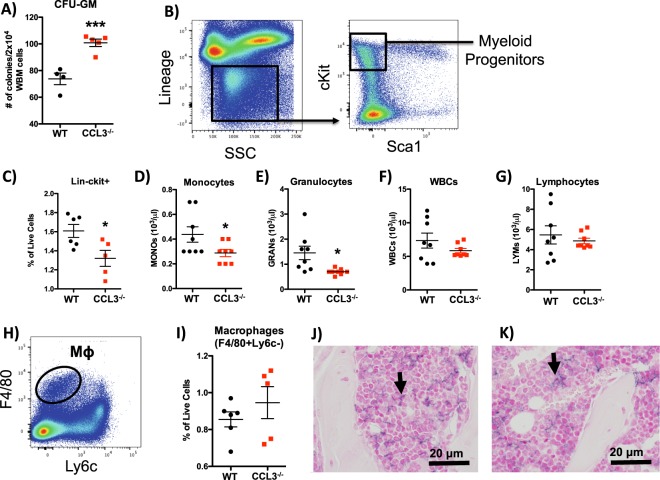
Myeloid cell differentiation is altered in CCL3^−/−^ mice. (**A**) Number of colonies counted following CFU-GM assay of bone marrow from WT and CCL3^−/−^ mice. (**B**) Schematic representation of flow cytometric analysis of bone marrow derived myeloid progenitors. (**C**) Quantification of bone marrow derived myeloid precursor cells depicted in B, reported as % of live bone marrow cells. Peripheral blood levels of (**D**) monocytes, (**E**) Granulocytes, (**F**) WBCs, and (**G**) Lymphocytes reported as number/μl of peripheral blood. **(H)** Schematic representation of flow cytometric analysis of bone marrow derived macrophages (MΦ). (**I**) Quantification of phenotypic MΦs depicted in H, reported as % of live cells. Immunohistochemical visualization of F4/80 in bone marrow of (**J**) WT and (**K**) CCL3^−/−^ mice. Each data point represents an individual mouse with mean and SEM depicted. Statistical significance determined by Student’s t-test *p ≤ 0.05, ***p ≤ 0.001.

CCL3 signaling has been shown to be critical for the maintenance and/or emergence of leukemia initiating cells in CML^12,22^. However, while previous reports have shown no overt hematopoietic abnormalities in CCL3^−/−^ mice a detailed analysis of HSC function in the absence of CCL3 signaling has not been performed. To perform an unbiased multidimensional analysis of the bone marrow we generated CyTOF data to phenotype bone marrow cells based on 31 antigens (Supplemental Table 3). This data was analyzed in an unbiased and multidimensional fashion using the tSNE algorithm. Analysis of the total bone marrow using tSNE revealed a similar distribution of phenotypic cell populations in CCL3^−/−^ mice as compared to WT controls (Fig. 1). However, one population of cells was consistently increased in CCL3^−/−^ mice, and further analysis of the phenotypic markers expressed by that specific population revealed that it was CD45+, negative for multiple markers of mature differentiated hematopoietic cells (lineage-), and positive for Sca1 and cKit (Fig. 1A,B). Further, when phenotypic lineage-Sca1+ cKit+ (LSK) cells, a population enriched for HSPCs, is superimposed on the tSNE plot LSK cells almost completely coincide with this population (Fig. 1C,D). In addition to alterations in HSPC populations, a larger population of cells demonstrates dramatic differences in distribution, and when the CD11b + CD2-B220-Ter119- population is superimposed on the tSNE plot of CyTOF data this population coincides with the larger differentially distributed population (Fig. 1E,F). This suggests that CCL3 regulates the distribution of myeloid cell populations under homeostatic conditions. CCL3 has previously been reported to regulate the recruitment and differentiation of myeloid lineage cells under inflammatory conditions but has not been described under homeostasis in the bone marrow^5^. Therefore, we further characterized myeloid differentiation in CCL3^−/−^ mice. In the marrow of CCL3^−/−^ mice we observed increased numbers of CFU-GM as measured by colony forming assays in methycellulose (Fig. 2A), but decreased myeloid progenitors as measured by flow cytometric analysis (Fig. 2B,C). In CCL3^−/−^ mice, peripheral blood monocytes and granulocytes are also decreased while peripheral total white blood cells and lymphocytes remain unchanged (Fig. 2D–G). Bone marrow macrophages were not changed in CCL3^−/−^ mice, as shown by flow cytometry (Fig. 2H,I) and by IHC (Fig. 2J,K). These data suggest that CCL3 is necessary for the maintenance and differentiation of myeloid progenitors in the bone marrow and in the regulation of myeloid cells in the peripheral blood but is not involved in the differentiation of bone marrow macrophages.

### CCL3 signaling regulates HSPC numbers in the bone marrow

**Figure 3:**
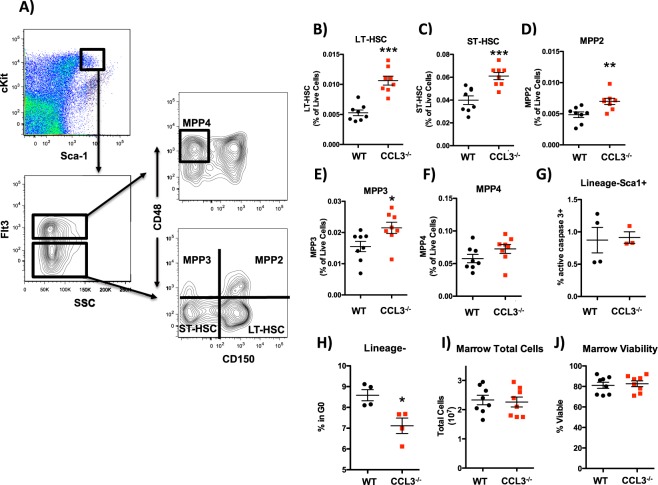
Increased number of phenotypic HSPCs in the marrow of CCL3^−/−^ mice. (**A**) schematic representation of phenotypic HSPC analysis of the bone marrow. (**B**–**F**) Quantification of LT-HSC, ST-HSC, MPP2, MPP3, and MPP4 populations depicted in (**A**). (**G**) Caspase 3+ cells reported as percent of lineage-Sca1+ HSPCs. (**H**) Ki67− 2n DNA content cells reported as percent of lineage-. (**I**) Total nucleated marrow cells per hindlimb. (**J**) Marrow viability. Each data point represents an individual mouse with mean and SEM depicted. Statistical significance determined by Student’s t-test *p ≤ 0.05, **p ≤ 0.01, ***p ≤ 0.001.

To confirm the CyTOF data and further analyze the HSPC population in CCL3^−/−^ mice and WT controls flow cytometric analysis was performed. This analysis revealed that CCL3^−/−^ mice have increased numbers of phenotypic HSPCs in the bone marrow compared to WT controls (Fig. 3A-F), extending previous reports that CCL3 acts to inhibit the proliferation of hematopoietic progenitors^17,18^ to include the HSPC pool. Populations that were increased included long-term repopulating HSCs (LT-HSC), short-term repopulating HSCs (ST-HSC), multipotent progenitor (MPP) 2, and MPP3, while the lymphoid biased MPP4 population was not increased (Fig. 3B–F)^23^. These data suggest that impairment of terminal myeloid differentiation in CCL3^−/−^ mice leads to increased myeloid biased progenitors, and that CCL3 regulates cell fate decisions between differentiation and self-renewal, of the most immature hematopoietic cells. While increases in HSPCs could be explained by a decrease in apoptotic rate, no change in rate of apoptosis was observed by measuring active caspase 3 by flow cytometry in the lineage- HSPC fraction of the bone marrow from CCL3^−/−^ mice and WT littermate controls (Fig. 3G). CCL3 has previously been reported to inhibit proliferation in hematopoietic progenitors^17^. Therefore we analyzed cell cycle status in the broad HSPC population by quantifying the frequency of actively cycling lineage- marrow cells that were ki67+. The frequency was increased in CCL3^−/−^ mice, indicating a greater percentage of HSPCs that were actively cycling (Fig. 3H). These data show that CCL3 acts to restrain the proliferation of HSPCs in the bone marrow. Since therapeutic treatment of many hematologic malignancies requires a functional HSPC pool to reestablish hematopoietic homeostasis, it is critical that treatment, including CCL3 inhibition avoids reduction of HSPCs within the bone marrow. These results demonstrate that phenotypically defined HSPC populations in the bone marrow are not decreased in the absence of CCL3 signaling and are, in fact, expanded in both frequency and total number, since bone marrow cellularity and viability are unchanged (Fig. 3I,J).

**Figure 4:**
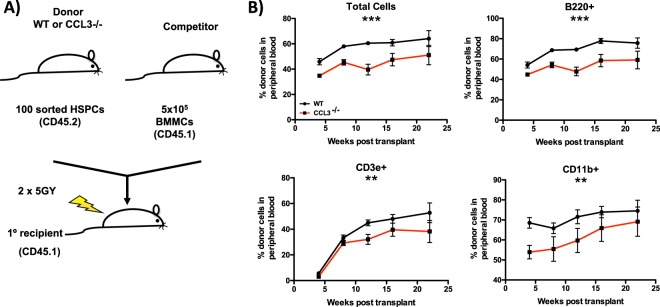
Decreased functional HSCs in CCL3^−/−^ mice. (**A**) Schematic representation of the competitive repopulation assay. (**B**) Longitudinal analysis of peripheral blood from primary transplant recipients to determine engraftment of sorted HSPCs from the bone marrow of WT or CCL3^−/−^ donor mice, n = 10 mice/group. Statistical significance determined by 2-way ANOVA. **p ≤ 0.01, ***p ≤ 0.001.

The increase in the number of phenotypic HSPCs in the bone marrow does not always correlate with increased HSPC function. Therefore, we sorted HSPCs from the marrow of WT and CCL3^−/−^ mice and performed competitive transplantation (Fig. 4A). In primary transplant recipients, CCL3^−/−^ HSPCs have reduced repopulating capability as compared to WT, and this difference was observed across myeloid, B-cell and T-cell lineages (Fig. 4B). These results demonstrate that CCL3^−/−^ mice maintain a functional yet relatively defective population of HSPCs capable of repopulating of the hematopoietic system.

### CCL3 does not regulate HSPC supportive BMME populations

**Figure 5:**
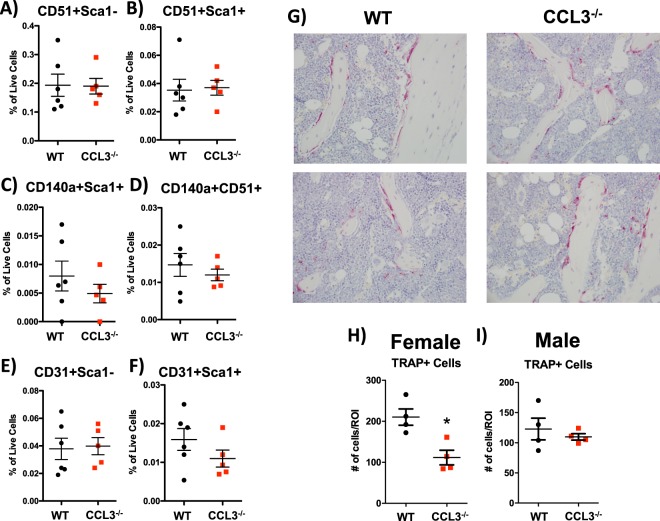
CCL3 does not regulate the HSC supportive BMME. Quantification of bone marrow osteoblastic cells (**A**), multiple populations of mesenchymal stem cells (**B**–**D**), arteriolar endothelial cells (**E**) and sinusoidal endothelial cells (**F**), data is reported as % of live bone marrow cells. (**G**) Histochemical analysis of TRAP activity on paraffin embedded sections in the bone marrow of WT and CCL3^−/−^ mice. Quantification of TRAP+ cells in (**H**) male and (**I**) female mice above the growth plate of the femur. Each data point represents an individual mouse with mean and SEM depicted. Statistical significance determined by Student’s t-test *p ≤ 0.05.

Hematopoietic stem and progenitor cells are regulated by their niche within the bone marrow microenvironment. The HSC niche is a complex network of molecular and cellular components^1^. Excess CCL3 in the setting of myelogenous leukemias and multiple myeloma has been shown to inhibit osteoblastic activity and activate osteoclastic bone resorption leading to overall loss of bone^6,7,24^. However, actions of CCL3 on bone during homeostatic bone remodeling have not been defined. Multiple cell populations that are critical for bone remodeling are also components of the HSC niche, and are negatively regulated by elevated levels of CCL3 in malignant conditions of the bone marrow, namely osteoblastic cells that form bone, and their precursors mesenchymal stem cells (MSCs)^6,7^. Under homeostatic conditions CCL3^−/−^ mice show no difference from controls in the number of Lineage-CD45-CD31-CD51+ Sca1- osteoblastic cells as measured by flow cytometric analysis (Fig. 5A)^24^. Additionally there was no significant difference in 3 MSC populations that have previously been described as supportive of HSCs, Lineage-CD45-CD31-CD51+ Sca1+ ^24^, Lineage-CD45-CD31-CD51+ CD140α+ ^25^, and Lineage-CD45-CD31-Sca1+ CD140α+ ^26^ (Fig. 5B–D). Endothelial cells are also supportive of HSCs within the niche and CCL3 has been described as exerting pro-neo-vascularization effects under malignant conditions^10^. However in CCL3^−/−^ mice we did not see any significant differences in sinusoidal Lineage-CD45-CD31+ Sca1- or arteriolar Lineage-CD45-CD31+ Sca1+ endothelial cells in the bone marrow (Fig. 5E,F).

Notably, when female and male mice were analyzed for TRAP + multinucleated osteoclastic cells in the bone marrow, we identified a significant decrease in osteoclasts in the bone marrow of female CCL3^−/−^ mice as compared to age and sex matched controls, while no difference was seen in male mice (Fig. 5I–K). This could be explained, in part, by a loss of monocyte osteoclastic precursors (Fig. 2).

**Figure 6:**
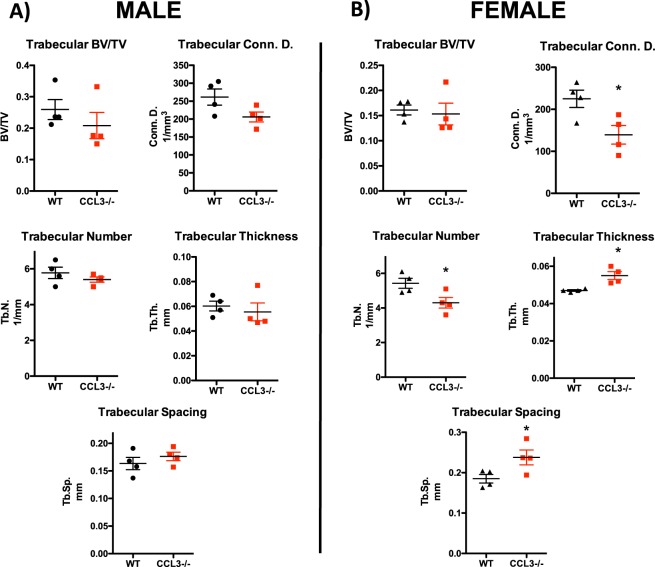
Reorganization of trabecular bone in female CCL3^−/−^ mice. Trabecular BV/TV, connectivity density, number, thickness, and spacing as measured by micro-CT in male (**A**) and female (**B**) mice. Each data point represents an individual mouse with mean and SEM depicted. Statistical significance determined by Student’s t-test *p ≤ 0.05, **p ≤ 0.01.

The difference in osteoclastic, but not osteoblastic, populations would be expected to lead to differences in bone architecture. Therefore, bone volume and microarchitecture was measured by μCT analysis. Male and female mice were analyzed separately due to sex specific differences in bone remodeling. In both sexes femoral trabecular bone volume normalized to total volume was unchanged in CCL3^−/−^ mice as compared to WT controls (Fig. 6A,B). As would be predicted by osteoclastic changes, female mice displayed differences in trabecular architecture, including a decrease in trabecular connectivity, and trabecular number, as well as an increase in trabecular thickness and spacing (Fig. 6A,B).

## Discussion

The chemokine CCL3 is relevant to human disease as a therapeutic target and previous findings in murine models, including our own, have demonstrated a role in hematologic malignancies^7,12,13,20,22^. The relevance to human disease is supported by our previous findings that CCL3 expression is elevated in the majority (~75%) of primary AML samples that were analyzed^7^. Therefore there is potential for translation of CCL3 pathway inhibition to the clinic. Additional support for the feasibility of CCL3 inhibition as a therapy for hematologic malignancies is provided by the availability of inhibitors for CCL3 receptors. CCL3 signals through the chemokine receptors CCR1 and CCR5^5^, and several small molecule antagonists have been developed for both of these receptors for a variety of diseases and with varying degrees of success^27^. CCR1 antagonism has been seen as a potential strategy for diseases such as rheumatoid arthritis and multiple sclerosis; however, clinical trials for the antagonists developed have generally failed in phase 2 after being found safe, but not efficacious for treatment^27^. Antagonists of CCR5 have also been developed for the treatment of HIV infection, as CCR5 is a co-receptor for cellular entry of HIV. One such antagonist, Maraviroc, was developed by Pfizer and is currently FDA approved for the treatment of HIV infection^28^. Notably, though CCL3 is emerging as a potential therapeutic target, its role in homeostatic regulation of HSCs has not been carefully studied.

To establish the role that CCL3 plays in regulation of homeostatic hematopoiesis and the BMME we characterized a global CCL3 knockout murine model. Results from flow cytometric analysis of HSPC populations demonstrate that lack of CCL3 signaling results in an expansion of the HSC pool in the bone marrow of CCL3^−/−^ mice. This is consistent with previous reports of CCL3 acting as an inhibitor of stem cell proliferation^17,18^. A careful analysis of the heterogeneous HSPC compartment in the bone marrow of CCL3^−/−^ and control mice revealed that in addition to HSCs, myeloid biased MPP2 and MPP3 populations were significantly increased, while lymphoid biased MPP4 populations were unchanged. Further, the levels of monocytes and granulocytes were lower in CCL3^−/−^ mice compared to WT. This indicates that CCL3 is involved in the differentiation process of these cell types, leading to a decrease of mature cells in the periphery and an increase in their progenitors in the bone marrow. Together these data suggest that CCL3 regulates the self-renewal capacity of the most immature hematopoietic cells, as well as the differentiation of progenitors of the myeloid lineage.

Increased numbers of phenotypic HSC populations in the bone marrow do not necessarily correlate with increased functional repopulating ability. For this reason we analyzed the ability of sorted HSPCs to repopulate the hematopoietic system of myeloablated recipients. Following competitive transplantation, CCL3^−/−^ HSPCs repopulate peripheral blood B cells, T cells, and myeloid cells with a reduced contribution compared to WT controls. These results suggest that increased proliferation and accumulation of HSPCs in the bone marrow is caused by a defect in myeloid differentiation and a relative loss of functional HSPCs in CCL3^−/−^ mice.

The differences that were observed can be caused either by direct signaling to HSPCs, or indirect signaling through alterations in the BMME. Micro-CT analysis of trabecular and cortical bone in the femur revealed no change in overall bone volume, though some minor differences in microarchitecture were observed in female mice, suggesting that CCL3 may regulate trabecular bone remodeling in a sexually dimorphic manner. Though it has previously been shown that CCL3 overexpression in malignant conditions leads to loss of mature osteoblastic cells, and over-activation of osteoclasts leading ultimately to a loss of bone^6,7^, loss of CCL3 expression has no effect on the osteoblastic populations, including MSCs. However, in female mice there is a loss of osteoclastic cells in the bone marrow, suggesting that the change in osteoclastic activity is the cause of altered trabecular architecture.

Overall, our data demonstrate that CCL3 plays an active role in the homeostatic maintenance of myeloid differentiation. Since CCL3 has previously been shown to be critical for the maintenance and emergence of leukemia initiating cells in multiple murine models, it will be important to evaluate any impact of CCL3 inhibition on the normal hematopoietic system as well as the malignancy being treated. However, these results, coupled with the availability of CCR1 and CCR5 antagonists suggest that the CCL3 signaling pathway may be an attractive therapeutic target in myelogenous leukemias.

## Materials and Methods

### Mice

Mice were maintained within the Vivarium facility at the University of Rochester School of Medicine and Dentistry in accordance with protocols approved by the University’s Institutional Animal Care and Use Committee, the University Committee on Animal Resources. Wild type C57BL/6J, and B6.129P2-*Ccl3*^tm1Unc^/J (CCL3^−/−^) mice fully backcrossed onto a C57BL/6J were purchased from Jackson Laboratory. B6.SJL-*Ptprc*^*a*^ *Pep3*^*b*^/BoyJ expressing the CD45.1 allotype were purchased from the mouse breeding core facility at the University of Rochester School of Medicine and Dentistry.

### Murine Cell Collection

Bone marrow cells were collected by flushing the long bones of the hind limbs of mice using a 25-gauge needle and FACS buffer (1x phosphate buffered saline supplemented with 2% fetal bovine serum). Peripheral blood cells were collected by submandibular bleeds into EDTA coated tubes. CBC counts were performed on a HESKA Hematrue. Peripheral blood white blood cells (WBCs) were collected by incubating blood in a 2% solution of 5 × 10^5^ molecular weight dextran to precipitate RBCs and the WBC containing supernatant was collected.

### Flow Cytometric Analysis, CyTOF, and Cell Sorting

For flow cytometric analysis 1 × 10^7^ cells were collected as described above and suspended in 100 μL PBS supplemented with 2% heat inactivated fetal calf serum (FACS buffer) and stained with the appropriate antibodies (Supplemental Tables 1 and 2). The cells were washed with 500 μL FACS buffer and data were collected on a LSR-II (BD Biosciences). The data were analyzed using FlowJo version 10.2 software (TreeStar). tSNE plugin was used on FlowJo. For sorting, cells were prepared as described for flow cytometric analysis and sorted using a FACSAria cell sorter (BD Biosciences) into FACS buffer.

For CyTOF analysis 10 × 10^6^ cells were collected as described and washed with 5 ml PBS. Cells were resuspended in 1 ml PBS with 5 μM cisplatin for live/dead discrimination and incubate at room temperature for 10 minutes. Cells were washed with 4 ml MaxPar staining buffer (DVS-Fluidigm cat# 201068), resuspended in PBS with 50 μM 127IdU cell identification reagent (DVS-Fluidigm cat# 201127) and incubated for 30 minutes at 37 °C. Cells were washed twice with MaxPar staining buffer, resuspended in 100 μl MaxPar staining buffer containing the appropriate extracellular antibodies (Supplemental Table 3), and incubated for 30 minutes at room temperature. Cells were washed with MaxPar staining buffer resuspended in 100 μl MaxPar staining buffer containing the appropriate secondary antibodies (Supplemental Table 3) and incubated for 30 minutes at room temperature. Cells were washed twice with MaxPar staining buffer, resuspended in 1 ml of 1x MaxPar Fix 1 Buffer (DVS Fluidigm cat# 201065) and incubated 15 minutes at room temperature. Cells were washed twice with 2 ml of MaxPar Perm-S buffer (DVS-Fluidigm cat# 201066) resuspended in 100 μl MaxPar fix and perm buffer (DVS Fluidigm cat# 201067) containing the appropriate intracellular antibodies and incubated for 30 minutes at room temperature. Cells were washed with MaxPar staining buffer resuspended in MaxPar Intercalator-Ir (DVS-Fluidigm cat#201192B) and incubated overnight at 4 °C. Cells were washed twice with MaxPar staining buffer, resuspended in deionized water at a concentration of 1 × 10^6^ cells/ml and analyzed on a CyTOF 2 mass cytometer (Fluidigm).

### Competitive Repopulation Assay

One hundred sorted LSK, Flt3- bone marrow cells from CCL3^−/−^ or C57bl/6 (CD45.2+) control mice were mixed with 5 × 10^5^ competitor whole bone marrow cells from B6.SJL-*Ptprc*^*a*^ *Pep3*^*b*^/BoyJ (CD45.1) mice in a total of 0.1 mL of FACS buffer. Recipient 6–8 week old B6.SJL-Ptprc^a^ Pep3^b^/BoyJ mice received a split dose of radiation of 5 Gy each separated by 24 hours. The second dose of radiation occurred 1–2 hours prior to injection of transplanted cells via tail vein.

### Micro-CT Analysis of Hindlimbs

Dissected hindlimbs were scanned on a Viva CT40 (Scanco Medical) using a 55-kVp, 145-μA current and a 300-ms integration time at a resolution of 12.5 μm. Analysis of trabecular bone was performed on a 1.25 mm region 50 μm below the growth plate of the femur and tibia. Cortical analysis was performed 4375 mm from the growth plate of the femur and tibia and encompassed a region of 375 μm.

### Histology and Immunohistochemistry

Dissected hindlimbs were fixed, decalcified, processed and embedded in paraffin as previously described^7^. Immunohistochemical staining for F4/80^29^ and Osteocalcin^30^ was performed as previously described.

## Electronic supplementary material

Supplementary information

## Data Availability

All data that support the reported findings are included in the manuscript, and are available from the corresponding author upon request.

## References

[1] Calvi LM, Link DC (2015). The hematopoietic stem cell niche in homeostasis and disease. Blood.

[2] Walkley CR (2007). A microenvironment-induced myeloproliferative syndrome caused by retinoic acid receptor gamma deficiency. Cell.

[3] Walkley CR, Shea JM, Sims NA, Purton LE, Orkin SH (2007). Rb regulates interactions between hematopoietic stem cells and their bone marrow microenvironment. Cell.

[4] Castellino F (2006). Chemokines enhance immunity by guiding naive CD8 + T cells to sites of CD4 + T cell-dendritic cell interaction. Nature.

[5] Menten P, Wuyts A, Van Damme J (2002). Macrophage inflammatory protein-1. Cytokine & growth factor reviews.

[6] Vallet S (2011). A novel role for CCL3 (MIP-1alpha) in myeloma-induced bone disease via osteocalcin downregulation and inhibition of osteoblast function. Leukemia.

[7] Frisch BJ (2012). Functional inhibition of osteoblastic cells in an *in vivo* mouse model of myeloid leukemia. Blood.

[8] Balderman S. R., Li A. J., Hoffman C. M., Frisch B. J., Goodman A. N., LaMere M. W., Georger M. A., Evans A. G., Liesveld J. L., Becker M. W., Calvi L. M. (2015). Targeting of the bone marrow microenvironment improves outcome in a murine model of myelodysplastic syndrome. Blood.

[9] Chen S (2016). Massive parallel RNA sequencing of highly purified mesenchymal elements in low-risk MDS reveals tissue-context-dependent activation of inflammatory programs. Leukemia.

[10] Wu Y, Li YY, Matsushima K, Baba T, Mukaida N (2008). CCL3-CCR5 axis regulates intratumoral accumulation of leukocytes and fibroblasts and promotes angiogenesis in murine lung metastasis process. Journal of immunology.

[11] Zucchetto A (2009). CD38/CD31, the CCL3 and CCL4 chemokines, and CD49d/vascular cell adhesion molecule-1 are interchained by sequential events sustaining chronic lymphocytic leukemia cell survival. Cancer research.

[12] Baba T (2013). MIP-1alpha/CCL3-mediated maintenance of leukemia-initiating cells in the initiation process of chronic myeloid leukemia. The Journal of experimental medicine.

[13] Dong L (2016). Leukaemogenic effects of Ptpn11 activating mutations in the stem cell microenvironment. Nature.

[14] Argiropoulos B (2008). Linkage of Meis1 leukemogenic activity to multiple downstream effectors including Trib2 and Ccl3. Experimental hematology.

[15] Horuk R (2009). Chemokine receptor antagonists: overcoming developmental hurdles. Nature reviews. Drug discovery.

[16] MacArthur RD, Novak RM (2008). Reviews of anti-infective agents: maraviroc: the first of a new class of antiretroviral agents. Clinical infectious diseases: an official publication of the Infectious Diseases Society of America.

[17] Graham GJ (1990). Identification and characterization of an inhibitor of haemopoietic stem cell proliferation. Nature.

[18] Broxmeyer HE (1990). Enhancing and suppressing effects of recombinant murine macrophage inflammatory proteins on colony formation *in vitro* by bone marrow myeloid progenitor cells. Blood.

[19] Broxmeyer HE (1989). Myelopoietic enhancing effects of murine macrophage inflammatory proteins 1 and 2 on colony formation *in vitro* by murine and human bone marrow granulocyte/macrophage progenitor cells. The Journal of experimental medicine.

[20] Dong L, Zheng H, Qu CK (2017). CCL3 is a key mediator for the leukemogenic effect of Ptpn11-activating mutations in the stem-cell microenvironment. Blood.

[21] Cook DN (1995). Requirement of MIP-1 alpha for an inflammatory response to viral infection. Science.

[22] Baba T (2016). MIP-1alpha/CCL3-expressing basophil-lineage cells drive the leukemic hematopoiesis of chronic myeloid leukemia in mice. Blood.

[23] Pietras EM (2015). Functionally Distinct Subsets of Lineage-Biased Multipotent Progenitors Control Blood Production in Normal and Regenerative Conditions. Cell stem cell.

[24] Schepers K (2013). Myeloproliferative neoplasia remodels the endosteal bone marrow niche into a self-reinforcing leukemic niche. Cell stem cell.

[25] Mendez-Ferrer S (2010). Mesenchymal and haematopoietic stem cells form a unique bone marrow niche. Nature.

[26] Morikawa S (2009). Prospective identification, isolation, and systemic transplantation of multipotent mesenchymal stem cells in murine bone marrow. The Journal of experimental medicine.

[27] Gladue RP, Brown MF, Zwillich SH (2010). CCR1 antagonists: what have we learned from clinical trials. Current topics in medicinal chemistry.

[28] Dorr P (2005). Maraviroc (UK-427,857), a potent, orally bioavailable, and selective small-molecule inhibitor of chemokine receptor CCR5 with broad-spectrum anti-human immunodeficiency virus type 1 activity. Antimicrobial agents and chemotherapy.

[29] Porter RL (2013). Prostaglandin E2 increases hematopoietic stem cell survival and accelerates hematopoietic recovery after radiation injury. Stem cells.

[30] Lawal RA (2017). The Notch Ligand Jagged1 Regulates the Osteoblastic Lineage by Maintaining the Osteoprogenitor Pool. Journal of bone and mineral research: the official journal of the American Society for Bone and Mineral Research.

