# Supplementary material for: The Chemokine CCL3 Regulates Myeloid Differentiation and Hematopoietic Stem Cell Numbers
Source: Sci Rep. 2018 Oct 2;8:14691. doi: 10.1038/s41598-018-32978-y (PMC6168534; doi:10.1038/s41598-018-32978-y)
Supplement: Supplementary file 1 — Supplementary information [file 41598_2018_32978_MOESM1_ESM.pdf]

The Chemokine CCL3 Regulates Myeloid Differentiation and Hematopoietic Stem Cell Numbers

Rhonda J. Staversky[1], Daniel K. Byun[1], Mary A. Georger[2], Brandon J. Zaffuto[2], Alexandra Goodman[1], Michael W. Becker[1,3], Laura M. Calvi[1,2,3,4], and Benjamin J. Frisch*[1,3,4]

[1]Department of Medicine Hematology/Oncology Division University of Rochester School of Medicine and Dentistry, Rochester, NY, USA
[2] Department of Medicine Endocrine Division University of Rochester School of Medicine and Dentistry, Rochester, NY, USA
[3] Wilmot Cancer Institute, University of Rochester School of Medicine and Dentistry, Rochester, NY, USA
[4] Center for musculoskeletal research, University of Rochester School of Medicine and Dentistry, Rochester, NY, USA

Supplemental Material:
Supplemental Table 1: Antibodies used for the flow cytometric analysis of bone marrow HSPCs.

Supplemental Table 2: Antibodies used for the flow cytometric analysis of engraftment in peripheral blood following competitive transplantation.

Supplemental Table 3: Antibodies used for the CyTOF analysis of the bone marrow microenvironment.

HSPC Flow Cytometry Antibodies			
Target	Clone	Fluorophore	Supplier and Cat #
Ter119	TER-119	Biotin (primary) PE-CF594-Streptavidin (secondary)	BD Biosciences; 560512
Gr1	RB6-8C5	Biotin (primary) PE-CF594-Streptavidin (secondary)	BD Biosciences; 561103
B220	RA3-6B2	Biotin (primary) PE-CF594-Streptavidin (secondary)	BD Biosciences; 561101
CD3e	145-2C11	Biotin (primary) PE-CF594-Streptavidin (secondary)	BD Biosciences; 551163
Sca1	D7	PerCP-Cy5.5	BD Biosciences; 558162
cKit	2B8	PE-Cy5	eBioscience; 15-1171
Flt3	A2F10	PE	eBioscience; 12-1351
CD48	HM48-1	FITC	eBioscience; 11-0481
CD150	9D1	APC	eBioscience; 17-1501

Supplemental Table 1: Antibodies used for the flow cytometric analysis of bone marrow HSPCs.

**Competitive Repopulation Antibodies**			
**Target**	**Clone**	**Fluorophore**	**Supplier and Cat #**
**B220**	**RA3-6B2**	**APC**	**BD Biosciences; 561872**
**CD3e**	**145-2C11**	**PerCP-Cy5.5**	**BD Biosciences; 561874**
**CD11b**	**M1/70**	**APC-Cy7**	**BD Biosciences; 561108**
**CD45.1**	**A20**	**PE**	**BD Biosciences; 561039**
**CD45.2**	**104**	**FITC**	**BD Biosciences; 561880**

Supplemental Table 2: Antibodies used for the flow cytometric analysis of engraftment in peripheral blood following competitive transplantation.

CyTOF Antibody Reagents			
Reagent	Format/Tag	Manufacturer	Catalog #
Anti-mouse CD140a	Purified	BD Pharmingen	562171
Anti-mouse CD2	Purified	BD Pharmingen	562182
Anti-mouse CD51	Biotin	BD Pharmingen	551380
Anti-mouse CD127 (IL7Rα)	Purified	Biolegend	135004
Anti-mouse Ly-6G/C (Gr-1) Antibody	141Pr	DVS-Fluidigm	3141005B
Anti-mouse CD41 Antibody	143Nd	DVS-Fluidigm	3143009B
Anti-mouse CD16/32 (FCGRII/III)	144Nd	DVS-Fluidigm	3144009B
Anti-β-Catenin	147Sm	DVS-Fluidigm	3147005A
Anti-mouse CD11b (Mac-1) Antibody	148Nd	DVS-Fluidigm	3148003B
Anti-mouse CD19 Antibody	149Sm	DVS-Fluidigm	3149002B
Anti-mouse CD25 Antibody	150Nd	DVS-Fluidigm	3150002B
Anti-mouse pAKT Antibody	152Sm	DVS-Fluidigm	3152005A
Anti-mouse TER119 Antibody	154Sm	DVS-Fluidigm	3154005B
Anti-mouse CD48 Antibody	156Gd	DVS-Fluidigm	3156012B
Anti-mouse CD45R (B220) Antibody	159Tb	DVS-Fluidigm	3159015B
Anti-p-PLCgamma2 [pY759]	162Dy	DVS-Fluidigm	3162018A
Anti-APC	163Dy	DVS-Fluidigm	3163001B
Anti-mouse Ly-6A/E (Sca-1) Antibody	164Dy	DVS-Fluidigm	3164005B
Anti-mouse CD31 (PECAM-1) Antibody	165Ho	DVS-Fluidigm	3165013B
Anti-mouse CD117 (Ckit) Antibody	166Er	DVS-Fluidigm	3166004B
Anti-mouse CD150 (SLAM) Antibody	167Er	DVS-Fluidigm	3167004B
Anti-Human (cross mouse) KI-67	168Er	DVS-Fluidigm	3168007B
Anti-mouse biotin antibody	170Er	DVS-Fluidigm	3170003C
Anti-mouse pS6 (S235/S236) Antibody	172Yb	DVS-Fluidigm	3172008A
Anti-FITC	174Yb	DVS-Fluidigm	3174006B
Anti-rabbit IgG Antibody	175Lu	DVS-Fluidigm	3175002G
Anti-mouse pCREB (Ser133) Antibody	176Yb	DVS-Fluidigm	3176005A
Anti-mouse CD135 (Flt-3)	APC	eBioscience	50-150-87
Anti-mouse CD34	Purified	eBioscience	16-0341
Anti-mouse CD105	Purified	R&D Systems	MAB1320
Anti-mouse CD45 Antibody	FITC	BD Pharmigen	561088

Supplemental Table 3: Antibodies used for the CyTOF analysis of the bone marrow microenvironment.
